# Study of Commercially Available *Lobelia chinensis* Products Using Bar-HRM Technology

**DOI:** 10.3389/fpls.2017.00351

**Published:** 2017-03-16

**Authors:** Wei Sun, Song Yan, Jingjian Li, Chao Xiong, Yuhua Shi, Lan Wu, Li Xiang, Bo Deng, Wei Ma, Shilin Chen

**Affiliations:** ^1^Key Laboratory of Beijing for Identification and Safety Evaluation of Chinese Medicine, Institute of Chinese Materia Medica, China Academy of Chinese Medical SciencesBeijing, China; ^2^Guangdong Provincial Key Laboratory of Applied Botany, South China Botanical Garden, Chinese Academy of SciencesGuangzhou, China; ^3^Pharmacy College, Heilongjiang University of Chinese MedicineHarbin, China; ^4^State Key Laboratory for Conservation and Utilization of Subtropical Agro-bioresources, South China Agricultural UniversityGuangzhou, China; ^5^College of Pharmacy, Hubei University of Chinese MedicineWuhan, China; ^6^Department of Oncology of Integrative Chinese and Western Medicine, China-Japan Friendship HospitalBeijing, China

**Keywords:** *Lobelia chinensis*, adulterants, Bar-HRM technology, ITS2, herbal medicine identification

## Abstract

There is an unmet need for herbal medicine identification using a fast, sensitive, and easy-to-use method that does not require complex infrastructure and well-trained technicians. For instance, the detection of adulterants in *Lobelia chinensis* herbal product has been challenging, since current detection technologies are not effective due to their own limits. High Resolution Melting (HRM) has emerged as a powerful new technology for clinical diagnosis, research in the food industry and in plant molecular biology, and this method has already highlighted the complexity of species identification. In this study, we developed a method of species specific detection of *L. chinensis* using HRM analysis combined with internal transcribed spacer 2. We then applied this method to commercial products purporting to contain *L*. *chinensis*. Our results demonstrated that HRM can differentiate *L. chinensis* from six common adulterants. HRM was proven to be a fast and accurate technique for testing the authenticity of *L. chinensis* in herbal products. Based on these results, a HRM approach for herbal authentication is provided.

## Introduction

*Lobelia chinensis* Lour. belongs to the family Campanulaceae, and is distributed widely in East Asian countries including China, Korea, and Japan (Tada et al., 1995). Various parts of the plant have been used for the treatment of snakebite, edema, diarrhea, and jaundice in Chinese folk medicine (Yang et al., 2014). Many active chemical compounds have been identified in the plant, including: piperidine alkaloids, such as lobeline, norlobelanine, and lobelanine; coumarins, such as 6,7-dimethoxycoumarin, 5-hydroxy-7-methoxycoumarin, and 5,7-dimethoxy-6-hydroxy-coumarin; and terpenoids, including phytol, phytenal, cycloeucalenol, and 24-methylene-cycloartanol (Ishimaru et al., 1992; Shibano et al., 2001; Yang et al., 2014). As interest in the clinical use of herbal materials has grown in recent years, the presence of adulterants in herbal health products has become more frequent (Han et al., 2016). Fraudulent labeling of this herbal product has become commonplace, giving rise to health concerns. Fraud control is therefore desirable as a method of supporting fair trade and safeguarding consumer health.

Numerous conventional methods—including morphological, microscopic, and chemical identification—have been used for species specific identification of this herbal medicine, with each method having particular limitations. The traditional identification is dependent on the knowledge of morphological characters that enable to differentiate species. However, the absence of diagnostic characters always decreases the reliability of results (Chen et al., 2014). DNA barcoding technology was developed to find universal and short region of DNA sequences for authenticating species in herbgenomics area (Chen and Song, 2016). Additionally, DNA-based methods, including High Resolution Melt analysis (HRM) with DNA barcoding have become popular as assays designed to detect the presence of adulterants (Kalivas et al., 2014; Buddhachat et al., 2015; Osathanunkul et al., 2015; Singtonat and Osathanunkul, 2015; Costa et al., 2016; Xanthopoulou et al., 2016). HRM analysis is based on the classic melt analysis of PCR fragments that allows genotyping and fingerprinting by discriminating DNA sequence variants such as single nucleotide polymorphisms (SNPs) and small insertion and deletions (indels) (Ririe et al., 1997; Reed and Wittwer, 2004; Palais et al., 2005; Hong et al., 2015). Improved instruments, able to make more measurements per unit time, and therefore per unit temperature decrease, together with new intercalating and saturating DNA dyes, are able measure PCR products’ melting behavior with very high resolution (Sun et al., 2016). We were able to develop the first HRM-based method capable of fine discrimination between different plant species in order to authenticate *L. chinensis*-based products and to identify adulterants.

Here, we present our results, and show that nuclear internal transcribed spacer 2 (ITS2) DNA barcoding coupled with HRM analysis is a very accurate method for the authentication of *L. chinensis* and its adulterants. This study features the development and use of a nuclear ITS2 gene region to reliably discriminate six species through the use of HRM assays.

## Materials and Methods

### Samples

Academic literature and field surveys of Chinese herbal medicine markets identify *Scutellaria barbata* D. Don., *Scutellaria indica* L., *Oldenlandia diffusa* (Willd.) Roxb. Hort. Beng. and *Mazus pumilus* (Burm. f.) Steenis and *Pratia nummularia* A. Br. et Aschers. as major adulterants of *L. chinensis* herbal products (Guo et al., 2016). Plant material for *L. chinensis, S. barbata, O. diffusa, M. pumilus, S. indica*, and *P. nummularia* used in this study was collected from the Hainan and Chongqing Branch of Institute of Medicinal Plant Development, Chinese Academy of Medical Sciences, and the Xishuangbanna Tropical Botanical Garden, Chinese Academy of Sciences. Identification of plant material was provided by the Institute of Chinese Materia Medica, China Academy of Chinese Medical Sciences. The samples were stored at herbarium which located in the Institute of Chinese Materia Medica, China Academy of Chinese Medical Sciences.

### DNA Extraction and HRM-PCR Amplification

Total genomic DNA was isolated from 10 mg dried leaf tissue using a Universal Genomic DNA Extraction kit (Tiangen Biotech, Beijing, China) following the manufacturer’s protocol. Final concentrations of all genomic DNA samples were adjusted to 50 ng/μL. The DNA was stored at -20°C for further use.

A conserved region of the ITS2 region of nuclear ribosomal DNA was chosen for the identification assay because of its low intraspecific variation and the fact that it has sufficient variability to distinguish between even closely related species (Yao et al., 2010). ITS2 universal primers (ITS2F: 5′-ATG CGA TAC TTG GTG TGA AT-3′ ITS3R: 5′-GAC GCT TCT CCA GAC TAC AAT-3′) were used in this study. HRM-PCR reactions were performed in a Rotor-Gene Q MDx instrument (Qiagen GmbH, Hilden, Germany). Reaction mixtures had a final volume of 25 μl, and contained 50 ng genomic DNA, 12.5 μL of 2 × HRM PCR master mix (Type-it HRMTM PCR Kit, Qiagen), 1 μL of 10 μM forward and reverse primers, and distilled water was added up to the final volume. Cycling conditions consisted of an initial denaturation step of 3 min at 94°C, followed by 40 cycles of 30 s at 94°C, 30 s at 56°C, and 45 s at 72°C. The fluorescent data for PCR amplification was recorded during the extension step on the green channel. The final melting step ramped from 70 to 95°C in 0.15°C increments with a 2 s hold time for each acquisition step. Fluorescent data were acquired at the end of each extension step during PCR cycles.

### HRM Data Analysis

Rotor-Gene Q software was used to analyze the melting profiles. The negative derivative of the fluorescence (*F*) over temperature (*T*) (d*F*/d*T*) curve displays the melting temperature (Tm), and the normalized raw curve depicts the decreasing fluorescence against increasing temperature. Genotypes of test samples were identified by selecting a representative sample for each species. Based on a confidence threshold of 90%, a confidence percentage for each genotype was calculated (Ganopoulos et al., 2012).

### Sequence Confirmation

The tested products that presented unmatched melting curves were directly sequenced using a 3730XL sequencer (Applied Biosystems, Foster, CA, USA). Proofreading and contig assembly of sequencing peak diagrams were performed using CodonCode Aligner V 3.7.1 (CodonCode Co., Centreville, MA, USA). All sequences were submitted to TCM Barcode (DNA Barcoding System for Identifying Herbal Medicine^1^) to identify unknown adulterants by BLAST.

## Results and Discussion

### Identification of *L. chinensis* Using HRM Analysis of the Universal ITS2 Barcoding Region

**FIGURE 1:**
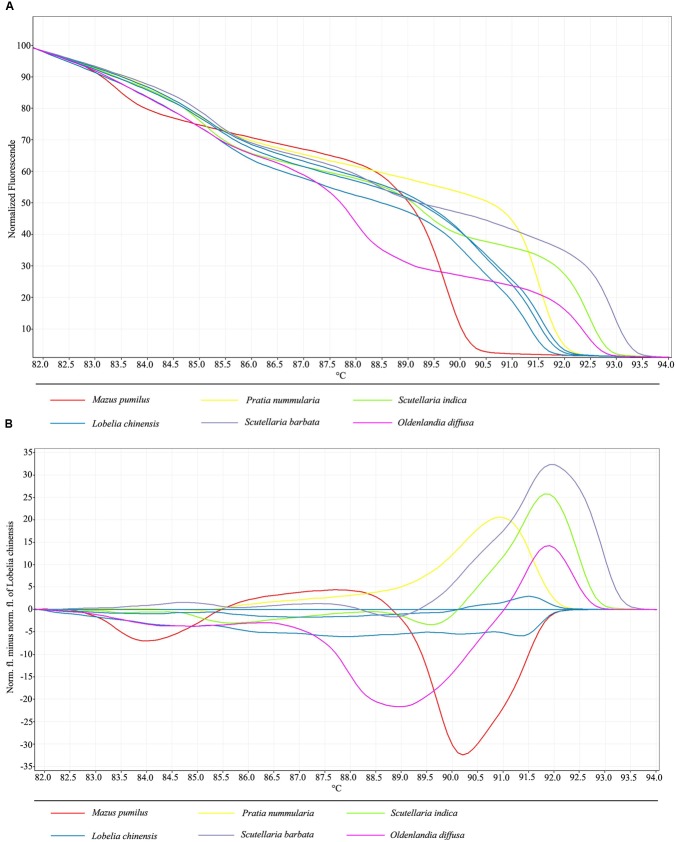
**(A)** High resolution melting analysis of all six species visualized as a graph of normalized melting curves. **(B)** High resolution melting analysis of all six species visualized as a difference graph using a haplotype of *L. chinensis* as the reference genotype.

In order to develop a fast method combining DNA barcoding and HRM analysis for discrimination of *L. chinensis* and its adulterant species, as well as to authenticate herbal products containing this species, HRM was performed on amplicons produced from primers for the nuclear ITS2 region. Normalized melting curves for the barcode marker ITS2 for six species are shown in **Figure 1**. Different herbal species tested did generate distinctive HRM profiles, allowing the discrimination and the differentiation of each species. Analysis of the normalized HRM curves produced with the ITS2 primer set revealed that all of the test species could easily be distinguished (**Figure 1A**).

In order to better visualize small differences between individual melting curves, HRM software applications allow calculation of a difference plot. By assigning *L. chinensis* as a genotype and using its melting curve as the baseline, by subtracting the melting curves of the other species, we were able generate difference data to estimate the similarity between the melt profiles of the ITS2 amplicons of *L. chinensis* and the five adulterant species tested in this study. Furthermore, genotype confidence percentages (GCPs) were calculated, with a cut-off value of 90% used to assign a genotype for each barcode region. With this approach, all samples were successfully genotyped, and the five adulterant species were specifically and confidently identified. Visualization and separation of variant melting curves is shown in **Figure 1B**. Furthermore, the HRM method permits use melting curves to discriminate between the three different haplotypes of *L. chinensis*, which differ by C/T base transposition at position 26 and A/T base deletion at positions 29 and 30; these results were validated by sequencing of amplified products. Thus, we conclude that HRM analysis of universal ITS2 amplicons is a powerful tool for the identification of *L. chinensis* and its adulterants.

### Identification of Plant Matter in Commercial Samples

**FIGURE 2:**
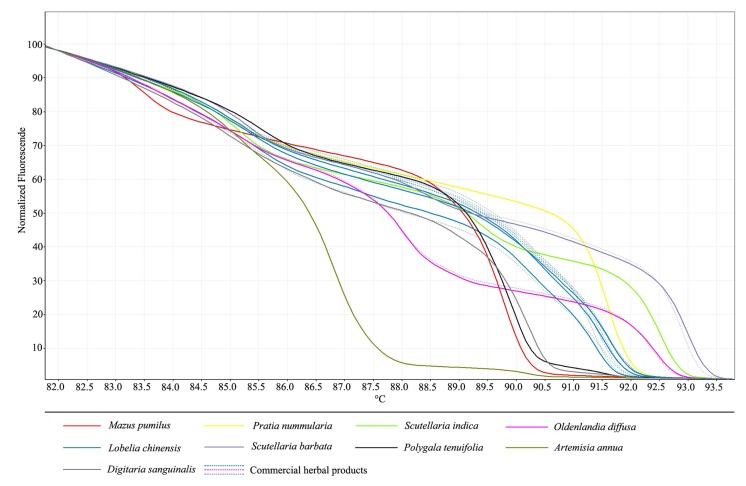
**High resolution melting analysis of 20 commercial medicinal materials visualized as a graph of normalized melting curves**.

After confirmation that all adulterant species can be identified by HRM analysis we used the same protocol to test for adulterants present in commercial herbal products. A total of 20 test samples were purchased from medicine markets, drug stores, and hospitals. All of samples were labeled as “*L. chinensis*,” were available to patients and consumers, and contained genomic DNA of sufficient quality and quantity for sequence amplification. Normalized HRM curves for ITS2 amplicons from these species in 20 commercial “*L. chinensis*” herbal products are shown in **Figure 2**. The melt analysis of the products of HRM-PCR amplification from *L. chinensis* gDNA showed three unique melting curves, due to intraspecific variation in the ITS2 sequence. However, out of 20 commercial samples, only 14 products contained *L. chinensis* as indicated; despite heavy processing, these samples produced melting curves and inflection shapes identical to those produced by ITS2 amplification of pure *L. chinensis* genomic DNA. The respective melting curve analyses revealed the amplification of similar products since they exhibited the same melting curve profile.

However, the melting curves of the other six products generated different melting profiles. Two tested samples were similar to *O. diffusa*, and one sample was consistent with *S. barbata*. In addition, the other three samples likely did not contain any *L. chinensis* whatsoever, but rather contained some other species. In order to find substituted species in these products, DNA barcoding is one of the best solutions. As can be seen from our previous works, DNA barcoding was performed to detect the adulteration and substitution of herbal drugs and found that herbal products sold on the markets were contaminated or substituted with alternative (often cheaper-to-produce) plant species that are not listed on ingredient labels. The ITS2 amplicons of these species did not correspond to the melt profiles of *L. chinensis* or any of the six tested adulterants; therefore, DNA sequencing of ITS2 regions was performed to identify species in these products. The BLAST result showed that these products have a sequences similar to those of *Polygala tenuifolia* Willd., *Artemisia annua* Linn., and *Digitaria sanguinalis* (L.) Scop., respectively. This finding provides evidence that aerial part without obvious characteristic from annual herbs are easily adulterated or substituted. Therefore, the plant substitution in *L. chinensis* herbal products sold in the Chinese local market, and may be a serious issue for consumers.

### Development of an Experimental Approach for Rapid Authentication of Herbal Medicine Using HRM Analysis

High Resolution Melting analysis has become a reliable and highly useful molecular technique in many fields, including infectious disease identification, food contaminant screening, and genotyping (Reed et al., 2007; Montgomery et al., 2010). HRM technology has been utilized for discriminating cultivated varieties, medicinal plant species, herbal tea, wine authenticity (Jung et al., 2010; Osathanunkul et al., 2015, 2016; Costa et al., 2016; Li et al., 2016; Song et al., 2016; Pereira et al., 2017). However, a reliable HRM approach to identify herbal medicines on Chinese herb market is still lacks. Designing a new HRM experiment involves sample collection, verification of voucher herbarium specimens, DNA extraction, experiment optimization, HRM amplification, melting profile analysis, and, finally, species identification and adulterant detection (Sun et al., 2016). First, it is crucial to be sure of the identity of the original plant used during the development of the HRM assay. The collections should be accompanied by photos and detailed field notes describing any identifying characteristics not evident from the herbarium specimens. Second, genomic DNA from both the authentic medicinal plant and the adulterants needs to be extracted and purified. The extracted DNA is then prepared for use as template for barcode-based PCR. Third, DNA barcode sequences are amplified with universal barcode primers (i.e., ITS2, *psb*A-*trn*H, *rbc*L, *mat*K, *rpo*C, *trn*L, etc.) or their combinations. Fourth, model melt curves should be constructed, both for the authentic herb and for common adulterants. Finally, the HRM assay system can be used to identify commercial herbal products. PCR product with one barcode can be used to sequence and blast for characterize herbal identity.

### The Potential and Limited Power of HRM Technology in Herbal Medicine Identification

At present, many herbal plant products distributed in Chinese local markets lack verification of the authenticity of their contents. Furthermore, as mentioned above, extracts containing *L. chinensis* have been contaminated with other low-cost species. These commercial herbal products, sold in local markets, occur in many different forms which lost obvious character for authentication. This variety makes accurate identification of the constituent species difficult. HRM screening permits discrimination of single nucleotide differences in DNA. In this study, HRM analysis was performed on amplicons from the ITS2 DNA barcoding locus, which has highly conserved nucleotide sequences at the species level. We used an HRM protocol to develop a single and fast test to determine the purity of *L. chinensis* herbal products, as well as to identify adulterant species. In previous studies, DNA barcoding has proven effective for detecting pure substitution in herbal medicines (Chen et al., 2010; Pang et al., 2013; Wu et al., 2015; Xin et al., 2015). However, it’s difficult to determine whether an unknown herbal product is mixed or pure constituent. Although cloning and next generation sequencing could provide the clues, we recommend HRM-based protocols may preliminarily detect their constitutes by analyzing melting curve. We conclude that HRM analysis is likely to become a routine test of herbal medicine quality and traceability. However, it is difficult to utilize HRM technology to differentiate species with small differences in meting temperatures (<0.25°C) and homozygotes with C to G and A to T changes in specific site (Simko, 2016). Another single barcode or multi-locus combination can provide optimized discrimination (Osathanunkul et al., 2016).

## Conclusion

This study was the first to use DNA barcoding coupled with HRM analysis to detect the species composition of *L. chinensis* herbal products currently on the market. When amplified using universal barcoding ITS2 primers, DNA extracted from all herbal specimen tested yielded specific amplification products. *L. chinensis* and its adulterants were easily distinguished by examining the HRM curves for these amplicons. Among 20 commercial herbal products sold in drug stores and markets in China as “*L. chinensis*,” we used HRM curves to confirm that while 14 tested samples were uncontaminated, six products were found to contain adulterant plant species. HRM technology was shown to be a fast and accurate closed tube post-PCR method which permits the identification of *L. chinensis* and its adulterants, thereby guaranteeing clinical drug safety and ensuring the vital interests of patients.

## Author Contributions

WS, SY, and JL finished experiments and data analyses. CX, LW, LX, YS, and BD contributed to collect materials. WM and SC designed this experiment.

## Conflict of Interest Statement

The authors declare that the research was conducted in the absence of any commercial or financial relationships that could be construed as a potential conflict of interest.

## Footnotes

1 http://www.tcmbarcode.cn/en/
